# Effect of Benzoylphenyl Ureas on Survival and Reproduction of the Lace Bug, *Leptopharsa gibbicarina*

**DOI:** 10.3390/insects12010034

**Published:** 2021-01-06

**Authors:** Luis Carlos Martínez, Angelica Plata-Rueda, José Eduardo Serrão

**Affiliations:** 1 Department of General Biology, Federal University of Viçosa, Viçosa 36570-000, Brazil; jeserrao@ufv.br; 2 Department de Entomology, Federal University of Viçosa, Viçosa 36570-000, Brazil; angelicaplata@yahoo.com.mx

**Keywords:** chemical insecticides, insect vector, oil palm, pest control, reproductive parameters

## Abstract

**Simple Summary:**

*Pestalotiopsis* fungal complex is a disease that causes damages in oil palm (*Elaeis guineensis*), and the lace bug, *Leptopharsa gibbicarina* is the main insect pest that spread this disease. Application of neurotoxic insecticides has been a common method used to control *L. gibbicarina* for decades in Colombia and Venezuela. The effects of four benzoylphenyl ureas (BPUs) (lufenuron, novaluron, teflubenzuron, and triflumuron) were assessed against *L. gibbicarina* for toxicity, survival, and reproduction. Overall, the results show that novaluron, teflubenzuron, and triflumuron cause high mortality and reduce survival time, fecundity, and fertility. Thus, BPUs exhibit detrimental effects on *L. gibbicarina* and can be used as alternatives to other chemical insecticides.

**Abstract:**

The lace bug, *Leptopharsa gibbicarina* is a vector of *Pestalotiopsis* fungal complex in oil palm crops in the Americas. The effects of four benzoylphenyl ureas (BPUs) (lufenuron, novaluron, teflubenzuron, and triflumuron) were evaluated against *L. gibbicarina* for toxicity, survival, reproduction, and mortality in semi-field conditions. Concentration-mortality bioassays demonstrated that novaluron (LC_50_ = 0.33 ppm), teflubenzuron (LC_50_ = 0.24 ppm), lufenuron (LC_50_ = 0.17 ppm), and triflumuron (LC_50_ = 0.42 ppm) are toxic to *L. gibbicarina* nymphs. The survival rate was 99% in control nymphs, decreasing to 50% in nymphs exposed to LC_50_ of triflumuron, 47% in nymphs treated with lufenuron, 43% in nymphs treated with teflubenzuron, and 43% in those treated with novaluron. Sublethal concentrations of BPUs showed detrimental effects on the adult emergence, longevity, fecundity, and fertility of this insect. The mortality of nymphs caused by these insecticides was similar in both laboratory and semi-field conditions. Our results suggest that novaluron, teflubenzuron, and triflumuron are highly effective against *L. gibbicarina*, and therefore, have potential applications for this oil palm pest.

## 1. Introduction

The lace bug, *Leptopharsa gibbicarina* Froeschner (Hemiptera: Tingidae) is a significant pest and main vector of the *Pestalotiopsis* leaf spot in oil palm (*Elaeis guineensis* Jacq. (Arecales: Arecaceae)) in Colombia and Venezuela. This insect damages other palm trees species, such as *Aiphanes horrida* (Jacquin) Burret, *Bactris gasipaes* (Kunth), and *Elaeis oleifera* (Kunth) [1]. The life cycle of *L. gibbicarina* is 69 days (egg = 15, nymph = 22, and adult = 32) [2]. This insect can reach high infestations in oil palms with different steps of the *Pestalotiopsis* fungal complex (*Pestalotiopsis palmarum* (Cooke) Steyaert and *Pestalotiopsis glandicola* (Castagne) Steyaert) evolution [3]. The severity of the *Pestalotiopsis* leaf spot disease seems to be due to the easy access given to the oil palm leaves by the piercing and sucking activities of *L. gibbicarina* [4,5].

In Colombia, chemical insecticides, such as deltamethrin, methamidophos, methyl parathion, and permethrin, are used on oil palm crops to control *L. gibbicarina* [6,7,8], but monocrotophos is the preferred compound, due to its reliably high efficacy [8]. Monocrotophos, a neurotoxic insecticide of the organophosphate group, is applied in oil palm trees by trunk injection or the root absorption method [9,10], and acts via ingestion by or contact with insects [9,10]. In commercial oil palm plantations, this insecticide is a hazardous compound, because residues have been found in crude oil [11]. Also, monocrotophos is banned in the European Union, United States, and several Latin American countries [12,13]. Alternatives that are more sustainable or different from monocrotophos are needed to substitute the principal insecticide used for the past 50 years against *L. gibbicarina* [6].

The application of chemical insecticides is an effective strategy for controlling pest populations [14,15,16], and the use of biorational insecticides is a valuable insect pest management option for oil palm plantations [17]. The current suite of biorational insecticides includes benzoylphenyl ureas (BPUs), characterized by biological activity interfering with developmental processes of insects [17]. In this context, BPUs and their effectiveness have also been reported to control oil palm pests like *Coptotermes curvignathus* Holmgren (Blattodea: Rhinotermitidae) in Malaysia [18], *Euprosterna elaeasa* Dyar (Lepidoptera: Limacodidae) in Colombia [19], and *Rhynchophorus ferrugineus* Olivier (Coleoptera: Curculionidae) in the United States [20]. The mode of action of BPUs remains elusive; however, evidence indicates that these insecticides inhibit the *N*-acetylglucosamine incorporation into insect chitin in vivo, altering of transport of proteins involved in chitin polymerization [21]. Especially, BPUs acts on immature insect stages (as ovicide or larvicide), with a broad spectrum against Diptera [22], Coleoptera [23], Hemiptera [24], Lepidoptera [25], and Neuroptera [26].

BPUs are chemical substance derivates of urea (H_2_NCONH_2_) [27] and are classified as inhibitors of chitin synthesis (affecting CHS1), according to the Insecticide Resistance Action Committee (IRAC, group 15) [28]. In particular, active ingredients like lufenuron, novaluron, teflubenzuron, and triflumuron are used successfully to control hemipterous disease vectors [24,29]. Non-neurotoxic insecticides like BPUs can be used against the oil palm pest, favoring an effective approach toward Integrated Resistance Management (IRM). There are a variety of insecticides that have neurotoxic properties used to control *L. gibbicarina*; however, the availability and use of biorational insecticides as BPUs is an alternative for pest management programs for oil palm. We hypothesized that the effects of BPUs reduce the number of nymphs and adults of *L. gibbicarina*, which could be due to its ability to inhibit chitin biosynthesis.

This research evaluated the insecticidal activity of four BPUs to control *L. gibbicarina*, explained in different experiments evaluating their (i) toxicity, (ii) survivorship, (iii) reproduction, and (iv) mortality in field conditions. Our objective was to contribute to the development of strategies for controlling *L. gibbicarina*, as the current main replacement for monocrotophos against this species.

## 2. Materials and Methods

### 2.1. Lace Bugs

In the field, adults of *L. gibbicarina* were collected from palm trees in Brisas Oil palm plantation (Puerto Wilches, State of Santander, Colombia), placed into plastic boxes (45 × 45 × 90 cm), and transported to the entomology laboratory to establish a colony in laboratory conditions. *Leptopharsa gibbicarina* adults (males and females, 1:1 ratio) were isolated in glass tubes (5 × 27 cm) containing *E. guineensis* leaflets. After female copulation, eggs oviposited on the surface of the leaflets were collected every 24 h and placed in glass tubes containing cotton wool saturated with distilled water. After hatching, nymphs were placed on a leaf of nursery oil palm tree (4 months old), isolated with an organza bag (45 × 90 cm), and maintained in a climatized room (27 ± 1 °C, 75–85% relative humidity, and light/dark 12:12 h cycle) until adult emergence. Newly third-instar nymphs and adults of *L. gibbicarina* were used in the laboratory and semi-field bioassays.

### 2.2. Concentration-Mortality Bioassay

The following commercial BPU formulations were diluted in 100 mL of deionized water to obtain six dilutions (ranging from 75 to 2400 ppm): lufenuron (Match EC, Syngenta Crop Protection S.A., Monthey, Swaziland), 50 g L^−1^; novaluron (Rimon EC, Makhteshim Chemical Works Ltd., Beer-Sheva, Israel), 100 g L^−1^; teflubenzuron (Dart SC, Dynamit Nobel GmbH, Leverkusen, Germany), 150 g L^−1^; and triflumuron (Alsystin SC, Bayer CropScience AG, Dormagen, Germany), 480 g L^−1^. Serial dilutions of each insecticide were used to assess toxicity and determine the concentration-mortality relationship and lethal concentrations (LC_25_, LC_50_, LC_75_, and LC_90_). Water alone was used as a control. Subsequently, each insecticide concentration (0.5 µL) was applied on the body of 50 third-instar *L. gibbicarina* nymphs using a Hamilton microsyringe (KH Hamilton Storage GmbH, Domat/Ems, Switzerland). The insects were individualized in glass tubes (1 × 12.5 cm) and maintained in a climatized room. A piece (1 × 9 cm) of *E. guineensis* leaf was provided daily as food before insecticide/control exposure. Three replicates of 50 nymphs were used for each concentration. The experimental design was completely randomized and the number of dead nymphs was recorded after 72 h of exposure.

### 2.3. Time-Mortality Bioassay

*Leptopharsa gibbicarina* nymphs were exposed to the lethal concentrations (LC_50_ and LC_25_) of each insecticide, as determined by the concentration-mortality bioassay. Water was used as a control. Exposure procedures and insect conditions were the same as described above (Section 2.2), with three replicates of 50 nymphs per treatment, following a completely randomized design. The number of live nymphs was counted every 6 h for 3 d.

### 2.4. Adult Emergence, Longevity, and Reproduction

*Leptopharsa gibbicarina* nymphs were exposed to lethal concentration (LC_25_) of each insecticide and monitored until adult emergence. In the control group, insects were exposed to water. The general maintenance of insects and plants were as described above. Newly emerged adults of *L. gibbicarina* were removed, sexed, and grouped into mating pairs. Each mating pair was then transferred to a single leaflet of a nursery oil palm tree of the same treatment and covered with organza fabric (5 × 50 cm) to prevent insect escape. Emergence and adult longevity was recorded every day until female/male death. Also, the number of eggs/female and number of nymphs/female hatched from these eggs were used to calculate fecundity and fertility, respectively.

### 2.5. Semi-Field Assays in Oil Palm Trees

The bioassay was conducted in five-year-old commercial oil palm plantations (varieties “Tenera” and “Deli Ghana”) in the county of Puerto Wilches (Santander, Colombia), with an average temperature of 27.59 °C, 76–89% relative humidity, 1490 to 2235 h of sunshine per year, and 2283 mm annual rainfall. Under these natural conditions, 50 palm trees were selected, and *L. gibbicarina* nymphs were used for each treatment in the controlled semi-field bioassay. For each palm tree, 50 nymphs were placed on leaf no. 17, according to the rules of phyllotaxy [30], and isolated with an organza bag (0.65 × 0.65 × 1.25 m) for 48 h to ensure natural insect distribution. Each insecticide was prepared to the LC_90_ level in water and used as treatments with five replications. Water was used as the control. Treatments were applied 48 h after placing the organza bag, and applications of 200 mL of each insecticide per leaf were made using a manual pump spray (Royal Condor, Soacha, Cundinamarca, Colombia; 1.8 L capacity) at 32 psi. Leaves from palm trees were cut with a Malay knife, carefully dissected, and checked for the presence of live or dead *L. gibbicarina* nymphs, which were then counted. For the treatment group on the cut leaf, *L. gibbicarina* mortality caused by insecticides was recorded every 15 d with a completely random experimental design.

### 2.6. Statistical Analysis

The concentration-mortality data were submitted to probit analysis to construct a concentration-mortality curve with the PROC PROBIT procedure (SAS Institute, Campus Drive Cary, NC, USA). Time-mortality bioassays were analyzed for survival analysis (Kaplan-Meier estimators, log-rank test) using the Prism 7.0 software (GraphPad Prism Software Inc., San Diego, CA, USA). Nymphs that remained alive at the end of the bioassay were censored for the analyses. Data on emergence, longevity, reproductive (fecundity and fertility) parameters, and field mortality of *L. gibbicarina* were subjected to one-way analysis of variance (ANOVA), with treatment as a fixed effect, and Tukey’s honest significance difference (HSD) test (*p* < 0.05) was used a mean separation test and analyzed with SAS 9.0 software. Data on emergence, longevity, reproduction, and field mortality test were arcsine-transformed to satisfy the premises of normality and homoscedasticity.

## 3. Results

### 3.1. Concentration-Mortality Bioassay

The concentration-response model used was suitable (*p* > 0.05), which confirmed the toxicity of each insecticide to *L. gibbicarina* and provided the estimates of the desired toxicological endpoints for subsequent use (Table 1). For the estimated LC_50_ value, testing indicated that novaluron with LC_50_ = 0.55 (0.36–0.74) ppm was the most effective BPU insecticide for *L. gibbicarina*, followed by teflubenzuron with LC_50_ = 1.71 (1.44–1.89) ppm, lufenuron with LC_50_ = 2.05 (1.78–2.33) ppm, and triflumuron with LC_50_ = 2.38 (2.07–2.71) ppm. In the control, mortality remained at <1%.

### 3.2. Time-Mortality Bioassay

**Figure 1:**
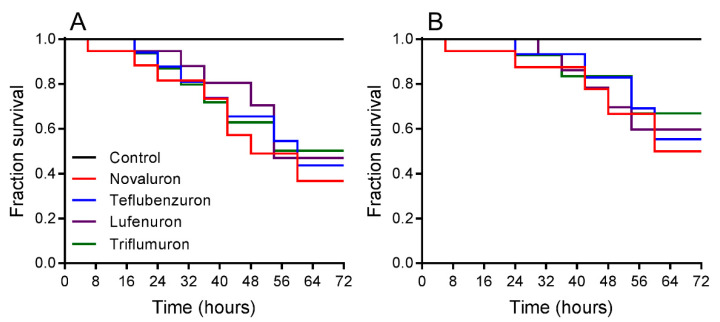
Survival curves of *Leptopharsa gibbicarina* nymphs exposed to four BPUs, estimated using the Kaplan-Meier log-rank test. Lethal concentrations: (**A**) LC_50_ (χ^2^ = 15.53, *p* < 0.0001) and (**B**) LC_25_ (χ^2^ = 8.94, *p* < 0.0012).

Survival rates were registered when *L. gibbicarina* nymphs were exposed for 3 d to BPUs and indicated differences at LC_50_ (log-rank test; χ^2^ = 15.53, df = 4, *p* < 0.0001) (Figure 1A). For the treatments, *L. gibbicarina* survival decreased from 99.9% in the control to 50.3% with triflumuron, 47.1% with lufenuron, 43.2% with teflubenzuron, and 36.7% with novaluron. Survival rates differed between treatments at LC_25_ (log-rank test; χ^2^ = 8.94, df = 4, and *p* < 0.0012). *Leptopharsa gibbicarina* survival decreased from 99.9% in the control to 66.9% with triflumuron, 59.7% with lufenuron, 55.3% with teflubenzuron, and 50.01% with novaluron (Figure 1B).

### 3.3. Adult Emergence, Longevity, and Reproduction

The effects caused by four BPUs on *L. gibbicarina* adults, such as emergence, survival, fecundity, and fertility, were determined (Table 2). The emergence of *L. gibbicarina* adults was different between the BPUs tested, with concentrations estimated for the LC_25_ values to females (*F*_4,19_ = 46.25, *p* < 0.0001) and males (*F*_4,19_ = 28.23, *p* < 0.0001). The longevity of adult *L. gibbicarina* decreased significantly when the insects were exposed to BPUs in females (*F*_4,19_ = 59.44, *p* < 0.0001) and males (*F*_4,19_ = 33.16, *p* < 0.0001). Similarly, the reproduction of this insect differed between the insecticides tested for fecundity (*F*_4,19_ = 11.68, *p* < 0.0001) and fertility (*F*_4,19_ = 21.62, *p* < 0.0001).

### 3.4. Semi-Field Assays in Palm Trees

**Figure 2:**
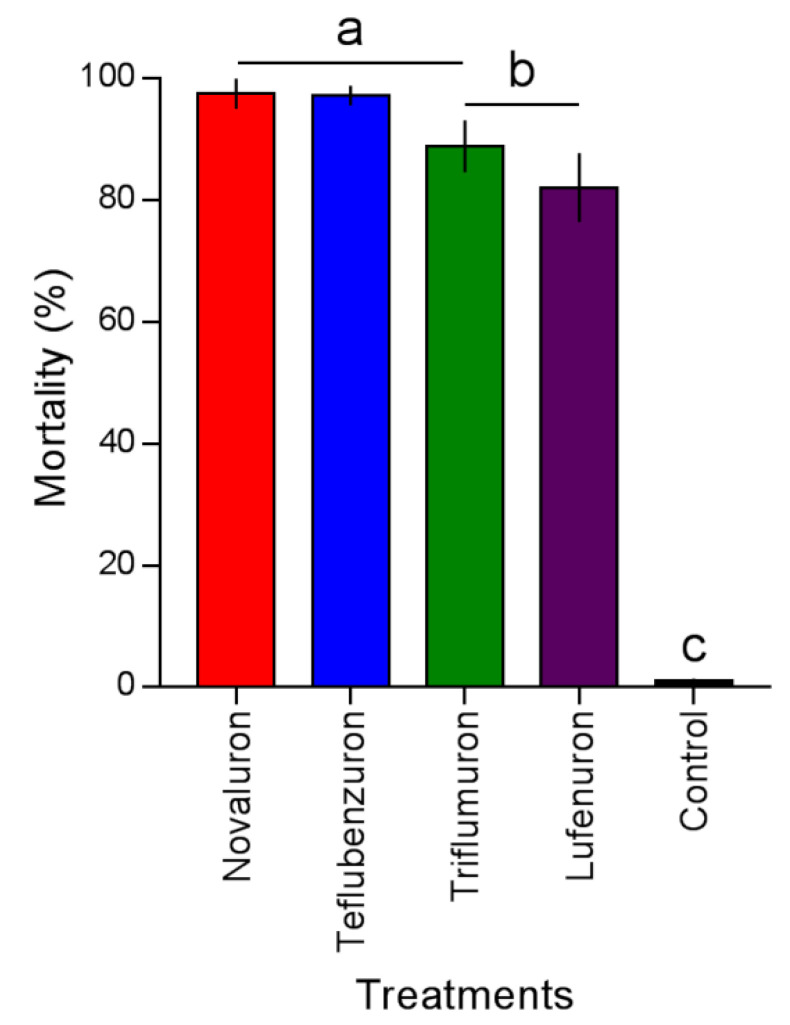
Mortality of *Leptopharsa gibbicarina* third-instar nymphs by four BPUs to level LC_90_ application on oil palm leaves. Treatment means (percent mortality ± SEM) with different letters show significant differences by Tukey’s HSD test at the *p* < 0.05 level.

The mortality caused by the BPUs on *L. gibbicarina* was different (*F*_4,9_ = 39.02; *p* < 0.0001) (Figure 2). Novaluron and teflubenzuron caused mortality of 97.5% ± 2.5% and 97.2% ± 1.6%, followed by triflumuron and lufenuron with 88.9% ± 2.7% and 82.1% ± 5.6%, respectively. Mortality did not exceed 1.04% ± 0.4% in the control.

## 4. Discussion

The use of various BPUs was effective in causing mortality, compromising survivorship, and affecting the reproduction of *L. gibbicarina* under laboratory and semi-field conditions. Novaluron, teflubenzuron, lufenuron, and triflumuron were toxic to *L. gibbicarina* nymphs and exerted a strong effect through contact exposure. BPUs caused mortality in *L. gibbicarina* in a concentration-dependent manner, as demonstrated in other insect vectors [24,29]. *Leptopharsa gibbicarina* nymphs exposed to high concentrations of BPUs displayed immobilization, cuticle malformation, and consequently, abortive molting. In this sense, symptoms in *L. gibbicarina* nymphs were consistent with the known effects of inhibitors of chitin synthesis. A set of results point to the effects on the cuticle of hemipteran pests, such as *Adelphocoris lineolatus* Goeze (Miridae) [30], *Aleurodicus rigioperculatus* (Aleyrodidae) [31], and *Stephanitys pyriodes* Scott (Tingidae) [32], after the topical application of BPUs. In general, BPUs exhibited toxicity against *L. gibbicarina* nymphs at different concentrations and reinforced their use as an alternative to neurotoxic insecticides on this species.

High variability in *L. gibbicarina* survival is mediated by the interaction of BPUs attaching to the external body surface and penetrating through the insect cuticle, leading to the suppression of ecdysis. The time taken for these BPUs to induce mortality in *L. gibbicarina* nymphs, from 48 to 72 h, presents quick action on this insect. In this study, the compared effects of BPUs on *L. gibbicarina* occur at various periods. These time differences occur due to BPUs’ ability to penetrate the integument cuticle layers [33], by changes in the proliferation of epidermal imaginal discs [34], and by altering the intracellular exocytosis process during chitin biosynthesis [35]. BPUs have been reported to induce nymph malformation, affect egg hatching, and interrupt the insect’s life cycle [32,36,37]. Low *L. gibbicarina* survival suggests that the insecticidal activity of novaluron, teflubenzuron, lufenuron, and triflumuron causes detrimental effects on nymphs, with an appreciable population reduction. Thus, they may represent a valuable alternative to monocrotophos and other pesticides to protect oil palm leaves.

The sublethal effect caused by the LC_25_ of each insecticide on the emergence, longevity, and adult reproduction of *L. gibbicarina* was observed. Exposure to novaluron, teflubenzuron, lufenuron, and triflumuron affects adult emergence, with a significant reduction in longevity. In this case, a low number of adults emerged as a result of the disruption of the reproductive cycle of this insect. Our results indicate that BPUs have the potential to suppress the development of *L. gibbicarina* populations, as observed in other studies [38,39]. With regard to reproduction, a smaller egg and nymph quantity was observed in the females of *L. gibbicarina* after novaluron, teflubenzuron, lufenuron, and triflumuron exposure. There are demonstrated ovicidal and nymphacidal activities in various hemipteran pests, such as *Agonoscena targionii* Lichtenstein (Aphalaridae) exposed to teflubenzuron [40], *Bagrada hilaris* Burmeister (Pentatomidae) exposed to novaluron [41], *Ceroplastes destructor* Newstead (Coccidae) exposed to triflumuron [42], and *Oxycarenus hyalinipennis* Costa (Lygaeidae) exposed to lufenuron [43]. The effects caused by BPUs on the fecundity and fertility of *L. gibbicarina* can be attributed to different changes during the embryonic developmental phase, compromising immature survival for various insects. Preliminary studies show that BPUs induce transovarial effects to produce a low number of eggs/nymphs when the insects are exposed during the adult stage [32] or before the adult emergence [44]. In this context, BPUs cause degeneration in the follicular epithelial cells of ovaries, reduction of vitellogenin deposits, distorted oocytes, and abnormal egg hatching [45]. The results suggest that BPUs have a high impact on the emergence, longevity, and reproduction of *L. gibbicarina*, affecting the fecundity, fertility, and offspring of this insect.

Novaluron, teflubenzuron, lufenuron, and triflumuron showed lethal effects against *L. gibbicarina* in palm trees in the field, and results were consistent with those observed in the laboratory. However, the mortality level at the nymphal stage was lower than those obtained under laboratory conditions. It is possible that the efficacy of BPUs in field conditions may be due to physical environmental factors [46], systemic or non-systemic action [47], chemical degradation [48], and limited persistence of insecticides in foliage [49]. However, while it is difficult to accurately determine the amount of insecticide penetrating to each insect, the mortality caused by these BPUs on *L. gibbicarina* was similar to trends observed for the application of insecticidal concentration. The lethality of BPUs and their effectiveness has also been studied with other oil palm pests under field conditions, proving them to be potent chemical agents against *Euprosterna elaeasa* Dyar (Lepidoptera: Limacodidae) exposed to teflubenzuron and triflumuron [50], as well as *R. ferrugineus* exposed to lufenuron and novaluron [51,52]. This was similar to findings that treating immature stages of *Drosophila suzukii* Matsumura (Diptera: Drosophilidae) with lufenuron in the United States [53], *Leptinotarsa decemlineata* Say (Coleoptera: Chrysomelidae) with novaluron in Canada [54], *Schistocerca gregaria* Forskal (Orthoptera: Acrididae) with teflubenzuron in Egypt [55], and *Spodoptera litura* Fabricius (Lepidoptera: Noctuidae) with triflumuron in Pakistan [56] reduced the population level of these pests. Our results show that BPUs have a specific physiological effect on insect growth that affects a high number of *L. gibbicarina* nymphs. In particular, novaluron, teflubenzuron, and triflumuron exhibit excellent insecticidal activity on this insect in the field, and the maximum efficiency from insecticides should be used during the nymph stage. Testing with these BPUs suggests that applications on oil palm leaves can drastically decrease *L. gibbicarina* infestation.

## 5. Conclusions

The side effects caused by four BPUs on the survival and reproduction of *L. gibbicarina* were investigated. Novaluron, teflubenzuron, lufenuron, and triflumuron inhibit the polymerization of chitin, cause mortality, and affect the reproduction of this insect, with the potential to control its field populations. The toxic effects of these insecticides may efficiently manage *L. gibbicarina* and reduce the insect’s damage and *Pestalotiopsis* fungal infection to oil palm leaves. In the field, *L. gibbicarina* was highly susceptible to novaluron, teflubenzuron, and triflumuron, and can be an alternative to monocrotophos in oil palm plantations.

## Figures and Tables

**Table 1:** Lethal concentration of four benzoylphenyl ureas (BPUs) against *Leptopharsa gibbicarina* nymphs after 72 h exposure, obtained from probit analysis (df = 5). The chi-square (χ^2^) value refers to the goodness of fit test at *p* > 0.05.

Insecticide	Lethal Concentration	Estimated Concentration (ppm)	95% Confidence Interval (ppm)	Slope ± SE	χ^2^ (*p*-Value)
Lufenuron	25	1.042	8.504–1.231	2.28 ± 0.16	3.70(0.44)
	50	2.054	1.789–2.339		
	75	4.049	3.528–4.742		
	90_0_	7.456	6.208–9.381		
Novaluron	25	0.221	0.112–0.342	2.93 ± 0.45	6.54(0.16)
	50	0.558	0.366–0.743		
	75	1.407	1.121–1.716		
	90_0_	3.233	2.604–4.298		
Teflubenzuron	25	0.778	0.597–0.958	1.96 ± 0.15	7.45(0.11)
	50	1.715	1.449–1.892		
	75	3.777	3.246–4.488		
	90_0_	7.689	6.264–9.983		
Triflumuron	25	1.181	0.966–1.392	2.21 ± 0.39	2.30(0.68)
	50	2.383	2.076–2.716		
	75	4.812	4.178–5.659		
	90_0_	9.055	7.501–1.145		

**Table 2:** Effects on the emergence, longevity, and reproduction of *Leptopharsa gibbicarina* caused by sublethal concentration (LC_25_) of the four BPUs. In the table, values followed with the same letter in the row do not differ significantly, according to the Tukey’s honest significance difference (HSD) test (*p* < 0.05).

Parameter	Control	Lufenuron	Novaluron	Teflubenzuron	Triflumuron
Female emergence (adults)	48.71 ± 0.76a	44.59 ± 1.08b	38.23 ± 1.23d	42.26 ± 0.55c	45.66 ± 0.67b
Male emergence (adults)	48.46 ± 0.61a	42.35 ± 1.03b	37.46 ± 1.32d	39.85 ± 0.49c	41.23 ± 0.32b
Female longevity (days)	36.81 ± 0.78a	30.79 ± 1.36c	24.76 ± 0.81d	29.28 ± 0.94c	33.82 ± 0.46b
Male longevity (days)	31.54 ± 0.78a	24.76 ± 0.81b	19.45 ± 0.81c	26.28 ± 0.87b	27.71 ± 1.62b
Fecundity (eggs/female)	86.65 ± 1.88a	72.30 ± 0.86c	58.15 ± 1.98d	68.85 ± 1.18c	79.80 ± 1.22b
Fertility (nymphs/female)	84.15 ± 0.97a	70.81 ± 0.87b	55.15 ± 1.32d	66.35 ± 0.96c	74.10 ± 0.49b

## Data Availability

Data sharing not applicable.
